# Energy-Aware Multipath Routing Scheme Based on Particle Swarm Optimization in Mobile Ad Hoc Networks

**DOI:** 10.1155/2015/284276

**Published:** 2015-12-24

**Authors:** Y. Harold Robinson, M. Rajaram

**Affiliations:** ^1^Department of Computer Science and Engineering, SCAD College of Engineering and Technology, Tirunelveli, Tamil Nadu 627414, India; ^2^Anna University, Chennai, Tamil Nadu 600 025, India

## Abstract

Mobile ad hoc network (MANET) is a collection of autonomous mobile nodes forming an ad hoc network without fixed infrastructure. Dynamic topology property of MANET may degrade the performance of the network. However, multipath selection is a great challenging task to improve the network lifetime. We proposed an energy-aware multipath routing scheme based on particle swarm optimization (EMPSO) that uses continuous time recurrent neural network (CTRNN) to solve optimization problems. CTRNN finds the optimal loop-free paths to solve link disjoint paths in a MANET. The CTRNN is used as an optimum path selection technique that produces a set of optimal paths between source and destination. In CTRNN, particle swarm optimization (PSO) method is primly used for training the RNN. The proposed scheme uses the reliability measures such as transmission cost, energy factor, and the optimal traffic ratio between source and destination to increase routing performance. In this scheme, optimal loop-free paths can be found using PSO to seek better link quality nodes in route discovery phase. PSO optimizes a problem by iteratively trying to get a better solution with regard to a measure of quality. The proposed scheme discovers multiple loop-free paths by using PSO technique.

## 1. Introduction

A MANET [1] is composed of mobile nodes connected by wireless media without centralized infrastructure. Routing schemes such as Dynamic Source Routing [2] and Ad Hoc On-Demand Distance Vector [3] were implemented to perform basic routing operation like forward data packets from a source to a destination. Routing schemes should consider the characteristics of the MANET. The prime needs of a MANET are reliability of data transmission, multipath selection [4], and providing security [5] which increases the network performance. Many of the researches have been developed to achieve this goal. On-demand routing [6] is one of the essential functions in MANET. The routing schemes [7] should be reliable, robust, and flexible in an ad hoc environment. Routing function is restricted by dynamic topology and link failure of the nodes. The mobility of the nodes increases the complexity of routing function because it causes of link failure between nodes. This frequent link failure leads to routing overhead and topology management, reduces the reliability of data transmission, and reduces the efficiency of the network. Hence, the link failure in MANET becomes a vital issue. Further, this sort of link failure also leads to frequent path failures. As a result, the reliability of data transmission gets reduced; the lesser the packet delivery ratio, the longer the end-to-end delay. Retransmission of data packets in a MANET is costly increasing the control message overhead and reducing the efficiency of the routing function. Hence, it is very much essential to select an alternate path or to form multiple paths for ensuring reliability of data transfer when a link failure occurs. And also loop-free path is very much interested in finding an optimal path among multiple paths between source and destination in a network.

Multipath routing in a MANET [8] is established in order to increase the reliability of data transmission that provides load balancing among the nodes. The use of multiple disjoint paths transferred the data in parallel that significantly increases the packet delivery ratio. Multipath routing schemes [9] deal with the problem of scalability, confidentiality, integrity, and network lifetime. Multiple-path routing [10] between source and destination ensures reliability of the data transmission in a MANET. Existing multipath routing schemes in a MANET lead to problems such as flooding, empty set of neighbors, flat addressing, widely distributed information, large energy consumption, interference, and load balancing issues. Therefore, the efficient multipath routing scheme is proposed to solve one or more of these issues. And also the existing multipath routing schemes do not perform well in dynamic environment change and frequent path failure. They also generate a routing overhead in the network. The routing overhead occupies a considerable portion of network bandwidth and the energy of the mobile node exhausts rapidly. Hence, with minimum overhead, the reliable multipath routing protocol is essential for designing to restrict the participation of mobile nodes in a route discovery phase that ensure reliability of data transmission. Evolutionary mechanism paradigm [11, 12] is most suitable to resolve multiobject problems because they are based on population. It generates a set of solutions in one run. There is no single solution that can be termed as the optimal solution [13] in multiobjective problems. In particle swarm optimization (PSO) [14, 15], the potential solutions of the problem are called particles. The group of particles becomes a swarm that searches for an optimum solution. Particle swarm optimization [16, 17] is a stochastic optimization technique in which the particles fly in the search space and adjust to the velocities dynamically according to their historical behaviors. This process guides the particles to soar toward the better search area in the search space [18]. It is difficult to ensure the reliability of the data transmission from source to destination because of its dynamism property and frequent link failure in MANET. The PSO can be applied to solve this kind of issue.

We design an energy-aware multipath routing scheme based on particle swarm optimization that uses continuous time recurrent neural network to solve optimization problems. Its goal is to discover multiple loop-free paths by using PSO technique. For gaps in the above literatures review, this work gives an optimum path selection technique that produces a set of optimal paths between source and destination to enhance the reliability of the data transmission.

The remainder of the paper contains five sections.  Section 2 summarizes previous research works.  Section 3 describes the proposed energy-aware multipath routing scheme based on particle swarm optimization technique in a MANET.  Section 4 presents our simulation results and a relevant performance analysis. Finally, Section 5 presents our conclusions and future direction.

## 2. Related Works

Evolutionary mechanisms [19] and clustering scheme [20] play a vital role to find optimal solutions for network partition. A number of evolutionary mechanisms have been proposed such as genetic algorithm [21, 22], artificial neural network system [23], particle swarm intelligence [24], and particle swarm optimization based clustering [25–27] that reveals the best global solutions. Dengiz et al. [28] proposed a particle swarm optimization algorithm that conceptualizes an autonomous topology optimization for mobile ad hoc networks using multiple mobile agents. The MANET communication is represented as network flows and optimization using a maximum flow model. This representation is very responsive to small changes in topology when evaluating network connectivity. Goswami et al. [29] proposed a fuzzy ant colony based routing protocol using fuzzy logic and swarm intelligence to select the optimal path by considering optimization of multiple objectives while retaining the advantages of swarm based intelligence algorithm. Ali et al. [30] proposed a multiobjective solution by using multiobjective particle swarm optimization algorithm to optimize the number of clusters in an ad hoc network and reduce the network traffic. This scheme performs intercluster and intracluster traffic that is managed by the cluster-heads. The authors considered the performance measures such as degree of nodes and power consumption of the mobile nodes.

Nasab et al. [31] proposed a multicast routing based on the particle swarm optimization (MPSO) that focused on efficient energy consumption and delay in multicast routing in MANET. It selects the node with the minimum energy consumption in the route selection and builds a multicast tree with minimum delay. There exists route failure in all route discovery methods resulting in data loss and routing overheads. Manickavelu and Vaidyanathan [32] proposed a Particle Swarm Optimization based Lifetime Prediction (PSOLP) algorithm for route recovery in a MANET. This scheme predicts the lifetime of link and node in the available bandwidth based on the parameters such as relative mobility of nodes and energy drain rate. The parameters are fuzzified and fuzzy rules have been formed to decide on the node status for prediction. Then, this piece of information is exchanged among all the nodes for verifying status of a node before data transmission.

Yun-Sheng et al. [33] proposed a multicast QoS based routing approach using genetic algorithm. It uses the available resources and minimum computation time in a dynamic environment. This scheme optimizes the routes by selecting the appropriate values for genetic operations like crossover, mutation, and population size. Radi et al. [34] proposed Low-Interference Energy-efficient Multipath Routing protocol (LIEMRO) to distribute source node traffic over the established paths. It also provides load balancing that estimates the optimal traffic rate of the paths. LIEMRO starts packet transmission immediately after the first path is established. Whenever a new path is created, the load balancing algorithm redistributes the source node traffic, according to the relative quality of the paths. Hurni and Braun [35] proposed a multipath routing protocol based on AOMDV. The objectives of these multipath schemes are to improve energy efficiency and reduce latency through load balancing and using cross-layer information. In order to reduce the end-to-end delay of data forwarding, each node utilizes the information provided by the MAC layer to transmit its packets to the neighboring node that wakes up earlier. Ghiasi and Karimi [36] proposed an algorithm of learning automata adjusting learning rate on neural network. It is a combination of the back-propagation algorithm, a local search algorithm, and learning automata to provide efficient global search. Mobile network parameters were measured for training and testing the neural network. The learning automata approach does not find optimal solution when the number of nodes increased in the network.

## 3. Energy-Aware Multipath Routing Scheme Based on Particle Swarm Optimization

MANET consists of mobile nodes with limited energy and wireless link. Each mobile node forwards the packets from source to destination. MANET is represented as directed graph *G* = (*V*, *E*). The vertices *v* ∈ *V* are a symbol of the mobile nodes and the neighbor node. An edge (*u*, *v*) ∈ *E* is a symbol of a wireless link between nodes *u*, *v*, which forward packets to others. The energy consumption for forwarding packets from a node *u* to node *v* is given by

(1) $$\begin{array}{c} E_{tx}\left(\;k,d\;\right)=E_{elec}\left(\;K\;\right)+E_{amp}\left(\;k,d\;\right), \end{array}$$

where *k* is number bits and *d* is the distance between nodes. *E*_elec_, *E*_amp_ are energy dissipated per bit to forward and receive packets, respectively. Energy consumption for receiver is calculated by

(2) $$\begin{array}{c} E_{rx}\left(\;k\;\right)=E_{elec}\left(\;K\;\right). \end{array}$$

The proposed EMPSO routing scheme is composed of three phases: (i) route setup phase, (ii) route discovery phase, and (iii) route maintenance phase. In the route setup phase, each node acquires its metadata of the neighborhood. This metadata is used in the route discovery to find the best next-hop node towards the destination node. The route discovery is activated whenever a source wants to transmit data to destination in an on-demand fashion that prevents multiple interference between source and destination. The route maintenance phase handles path failures during data transmission.

### 3.1. Route Setup Phase

In the route setup phase, source node initiates a data transmission for forwarding packets to the destination. Each node in a MANET obtains its metadata of the neighborhood, which also includes the transmission cost (*t*_*c*_) of its neighbors towards the destination node. The *t*_*c*_ value of a link indicates the required number of transmissions for a successful packet reception at the receiver. The transmission cost of a link is given as follows:

(3) $$\begin{array}{c} t_{c}=\frac{1}{p\times q}, \end{array}$$

where *p* and *q* are the probabilities of forward and backward packet reception over a link, respectively. In the initialization phase, each node broadcasts the control packets and stores the number of successfully received packets from its neighbors in the routing neighborhood table. Then, the destination node sets its transmission cost to zero and broadcasts this value to its neighbors, when a node receives a transmission cost included in a packet.

### 3.2. Route Discovery Phase

Whenever a source node wants to transmit data to destination, the route discovery phase is initiated to find multiple paths from the source to destination. The proposed multipath routing protocol uses reliability measures such as transmission cost, optimal traffic ratio, and remaining energy. The source node starts the route discovery by transmitting a route request packet (RR) towards the destination node. Whenever an intermediate node receives a RR packet, it computes the transmission cost, optimal traffic ratio, and remaining energy for a path that is established between the source and the destination. Then, it also used a found path to forward the RR packet to the neighboring node with minimum cost. The reliability measures are stored in the routing table of a node in MANET. The proposed EMPSO scheme uses a continuous time recurrent neural network to find an optimal path among multiple paths. CTRNNs are more computationally efficient in order to use a system of ordinary differential equations to model. The three weight factors such as transmission cost, energy factor, and optimal traffic ratio are taken into the account in CTRNN to find an optimal path. The weight factor of transmission cost is given in

(4) $$\begin{array}{c} w_{tc}={CR}_{pkt}\left(\;t_{i,j}\;\right)+{DR}_{pkt}\left(\;t_{i,j}\;\right), \end{array}$$

where *w*_tc_ is the weight factor for transmission cost, CR_pkt  _ is the control packet transmission ratio and DR_pkt_ is the data packet transmission ratio from node *I* to node *j*, respectively, and *t*_*i*,*j*_ is the time taken for CR_pkt  _ and DR_pkt_. The weight factor for optimal path ratio and remaining energy of a node are calculated as in

(5) wopr=1pk∑f=1n1/pf,wRE=ET−ETX+ERX+Eideal.

For a neuron *i* in the network with action potential *y*_*i*_ the rate of change of activation is given in

(6) Tiyi=−yi+σ∑i=1nwtcyi+∑i=1nwopryi+∑i=1nwREyi−θj+Iit.

Notations used in (5) and (6) are as follows:

 
*p*_*k*_: *k*th path;
 
*E*_*T*_: total energy required for packet forwarding from node *I* to node *j*;
 
*E*_TX_, *E*_RX_, *E*_ideal_: transmitted energy, received energy, and ideal energy of a node, respectively;
 
*T*_*i*_: time constant of postsynaptic node;
 
*y*_*i*_: rate of change of activation of postsynaptic node;
 
*w*_tc_: weight vector of transmission cost from presynaptic to postsynaptic node;
 
*w*_opr_: weight vector of optimal path ratio from presynaptic to postsynaptic node;
 
*w*_RE_: weight vector of remaining energy of node;
 
*σ*(*x*): sigmoid of *x*;
 
*θ*_*j*_: bias of presynaptic node;
 
*I*_*i*_(*t*): input to node.

**Figure 1:**
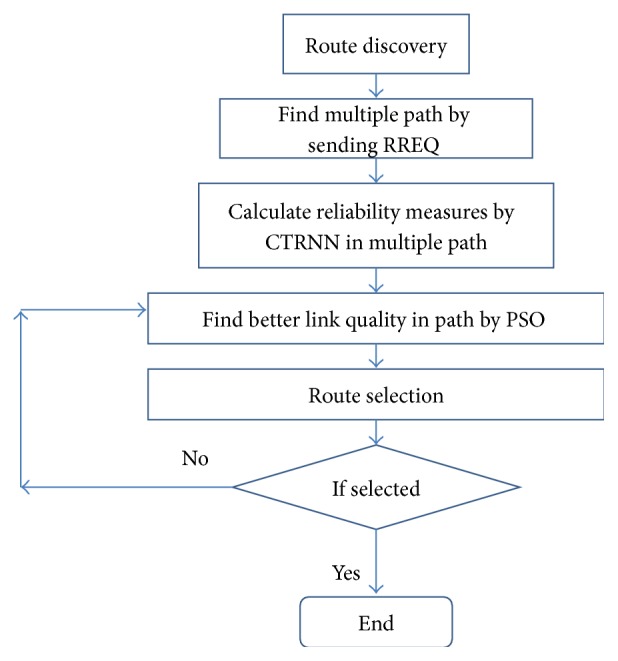
Flow diagram of the proposed system.

Figure 1 shows the flow diagram of the proposed scheme. Initially, multiple paths are found by route discovery phase. Then, reliability measures for a path are calculated with help of CRRNN. An optimization technique called PSO is used to find better link quality in a path. PSO can be applied to optimization problems that are partially in dynamic topology changing environment. PSO is an evolutionary optimization technique that may be used to seek a good set of weights in CTRNN. PSO is applied to find the best nodes (particles) involved in a path. PSO is metaheuristic that searches large spaces of candidate solutions. A route with a better link quality is selected for forwarding data from source to destination. If a better link quality is not found, PSO function is performed again until global best solution has been found. PSO reduces the traffic and routing overhead of the optimization process and finds the node with best link quality in an ad hoc network.

### 3.3. PSO Algorithm

Initialize nodes (particle) in a MANET.

For particle *i*, which is at distance *x*(*i*)

 Choose a path *p* of *N* particles among multiple paths.
 Find *fbest*(*p*), the best objective function among the neighbors, and global best *g*(*p*), the distance of the neighbor with the best objective function.
 Initialize the particle's position with a uniformly distributed random vector (*u*_1_, *u*_2_)
  
*v* = *W∗v* + *y*_1_ *∗u*_1_ *∗*(*p* − *x*) + *y*_2_ *∗u*_2_ *∗*(*g* − *x*).
 This update uses a weighted sum of the following:
  (i) The previous velocity *v* is found by speed of a packet
  (ii) The difference between the current distance and the best distance the particle has seen *p* − *x*
  (iii) The difference between the current distance and the best distance in the current neighborhood *g* − *x*
 
*x* = *x* + *v* // Update the distance
 Enforce the bounds. If any component of *x* is outside a bound, set it equal to that bound.
 
*f* = fun(*x*) // Evaluate the objective function
 If *f* < fun(*p*), then
   
*p* = *x*.
 If *f* < *b*, then
   
*b* = *f* and *d* = *x*.
 
*g* = *p*
 
*g* holds the best found solution
 If in the previous step or the best function value was lowered, then
   flag = true.
 Otherwise,
   flag = false // The value of flag is used in the next step.
 Update the neighborhood.
 If flag = true
   
*c* = max⁡(0, *c* − 1).
   minNeighborhoodSize = *N*
   If *c* < 2, then
     
*W* = 2*∗W*.
   If *c* > 5, then
     
*W* = *W*/2 // Ensure that *W* is in the bounds of the Inertial Range option.
 If flag = false:
   
*c* = *c* + 1.
   
*N* = min(*N* + minNeighborhoodSize, SwarmSize).

PSO is initialized with a group of particles and then searches for an optimal candidate solution by updating generations. Each particle is updated by two best values in the iterations. The first one is the best solution that has been achieved previously. The second best value is tracked by the particle swarm optimizer obtained currently by any particle in the population. The bound of the inertial range option is used for providing a satisfactory solution that eventually is discovered. This best value is a global best. The PSO algorithm significantly reduces the traffic overhead and computation complexity. The proposed PSO scheme reduced the route failure between nodes that minimize the routing overhead. To decrease the effect of random error, every experiment repeats 50 times and the average of experimental results is used as the performance metrics.

## 4. Results and Discussion

The proposed scheme has been implemented in a network simulator (NS2). The main objective of the simulation was to ensure reliability during data transmission in routing. The parameter settings are listed in Table 1. Nodes were randomly deployed in a 1000 m × 1000 m area of interest. The transmission range was 20 m. Nodes followed the random waypoint model that finds the availability of connection paths in a MANET. The performance of the proposed scheme was evaluated by comparing it with the related PSOLP and MPSO schemes in terms of packet delivery ratio, routing overhead, latency, energy consumption, and path optimality. The simulation results were studied by varying the network size from 50 to 200. The proposed scheme has integrated the PSO and continuous time recurrent neural network to enhance energy efficiency and reliability by selecting an optimal path. With the objective of comparing routing performance with related approaches, the proposed scheme has modeled a PSO in terms of functionality for reliable routing.

### 4.1. Performance Metrics

The proposed scheme uses five performance metrics to evaluate the proposed scheme and related schemes.

*Packet Delivery Ratio*. It is the ratio of the number of data packets received successfully by the destination node. 

*Latency*. It is the average time taken by the data packets sent from source node to the destination node. 

*Routing Overhead.* The number of control packets was generated during data transmission in routing. 

*Path Optimality.* It is the ratio of the total number of hops in the shortest paths to the total number of hops in the paths taken by the data packets. 

*Energy Consumption.* It is the average energy consumed for the data transmission in routing.

### 4.2. Packet Delivery Ratio

**Figure 2:**
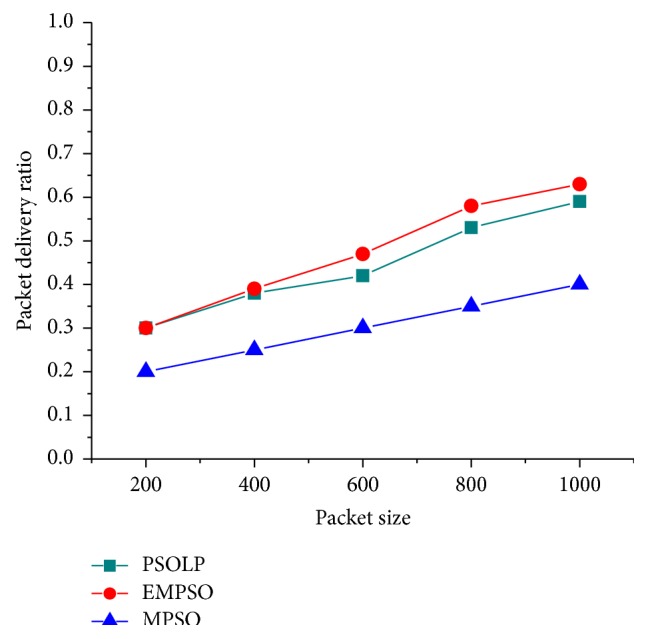
Packet delivery ratio versus packet size.

**Figure 3:**
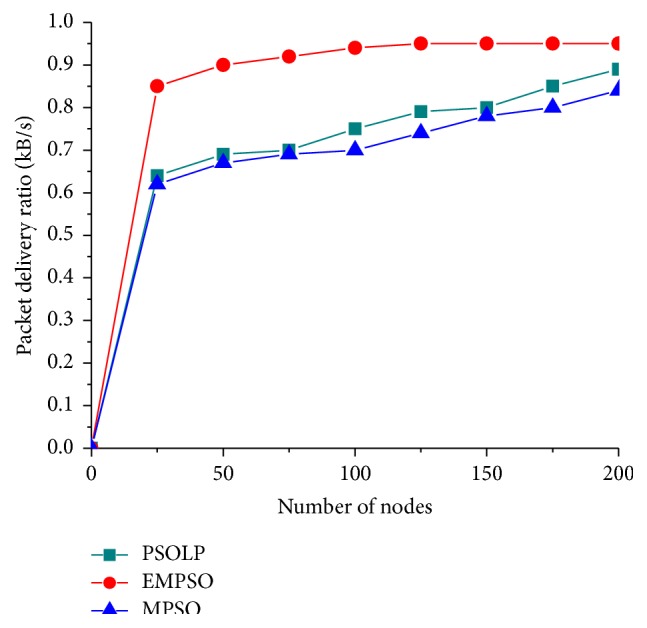
Packet delivery ratio versus number of nodes.

**Figure 4:**
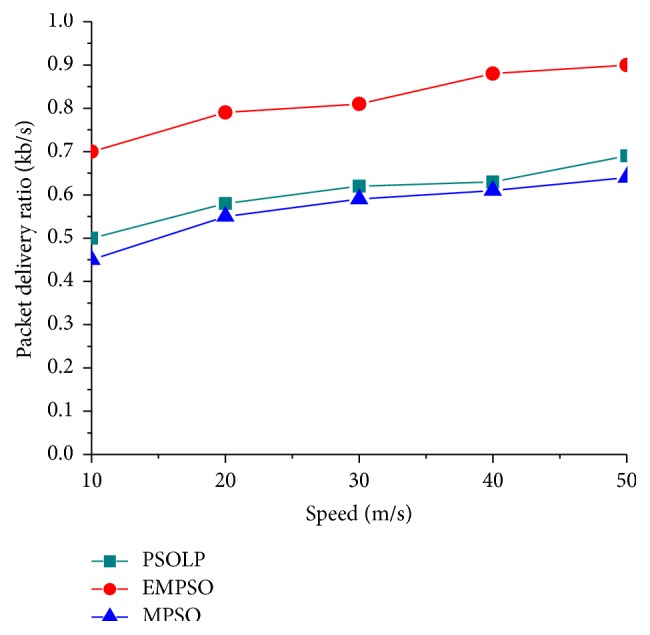
Packet delivery ratio versus speed.

In this simulation, the impacts of mobility, packet size, and network size were evaluated while measuring the PDR. It shows that the proposed schemes maintained higher PDR about 63%, while varying packet size increases. An intensive performance evaluation shows that the proposed scheme has better capability of finding an optimal route with the help of PSO approach.  Figure 4 shows that the PDR of the proposed schemes gets increased when the number of nodes increases. It shows that the packet delivery ratio increases for the proposed model since it provides the multipath routing when compared with the existing schemes. It was clearly shown that the performance of the proposed scheme is more efficient than the related schemes. Normally, the value of PDR gets increased in the proposed model since it sends the number of data at a time when compared to the related schemes.  Figure 3 showed that the PDR increases since it provides multipath routing with the best path. The other two approaches have no effective mechanism to find optimal path for routing.  Figure 2 shows that the proposed schemes maintained higher PDR about 90% in mobility scenario. PSOLP and MPSO have a smaller PDR because they have no effective routing mechanism to find optimal path among multiple paths.

### 4.3. Latency

**Figure 5:**
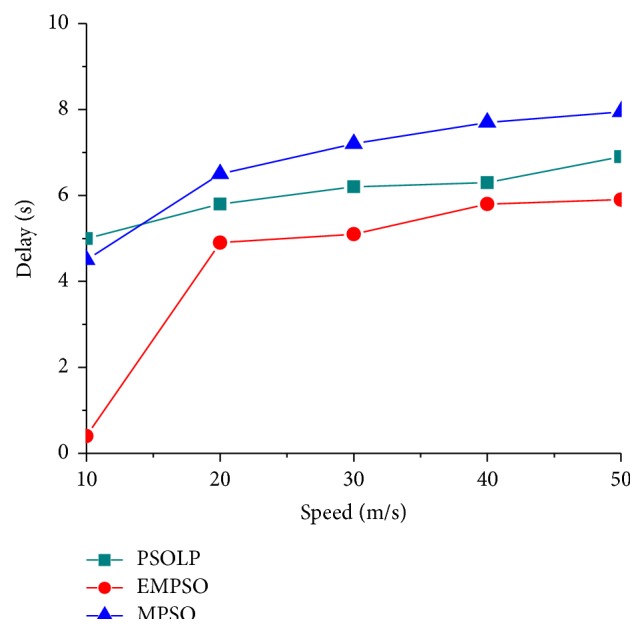
Delay versus speed.

**Figure 6:**
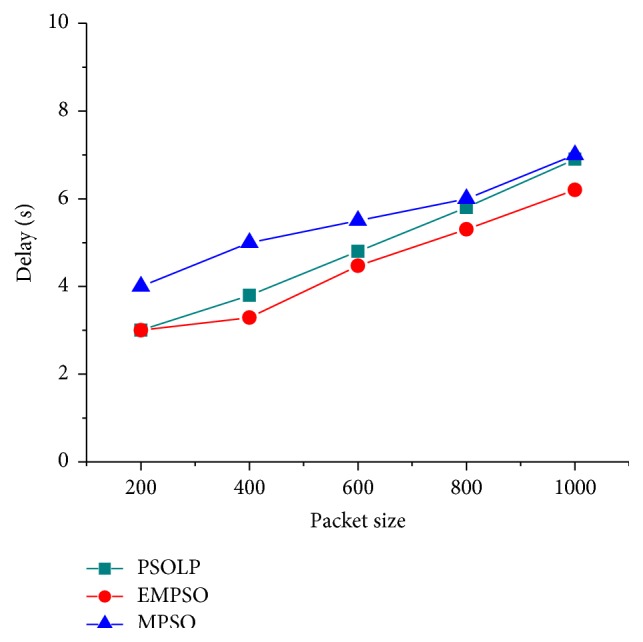
Delay versus packet size.

Figure 5 shows the delays of EMPSO, PSOLP, and MPSO measured from the simulation. It has been seen that the delay occurred for both the techniques when the speed is increased from 10 to 50 m/s. The delay begins to increase when the speed is increased since the chances of route breakage are more at high speed. Since the proposed EMPSO scheme predicts link quality more accurately than PSOLP and MPSO, the delay is 0.3% less for the related scheme. It was determined that the performance of the proposed scheme is more efficient than the related PSOLP and MPSO schemes.  Figure 6 shows the routing latency for three protocols when the number of packets varied. It was observed that the routing latency of the proposed EMPSO is 5.3 sec with multipath scenario on the network and 7 sec and 8.2 sec for PSOLP and MPSO, respectively. The related PSOLP and MPSO schemes took more time to forward packets to their destinations. The delays in both schemes are because of link failures and more exchanges of control messages in the routing. The proposed EMPSO scheme uses reliability measures to form multipath in a network. Therefore, it provides reliable routing and enhances the performance of MANET.

### 4.4. Routing Overhead

**Figure 7:**
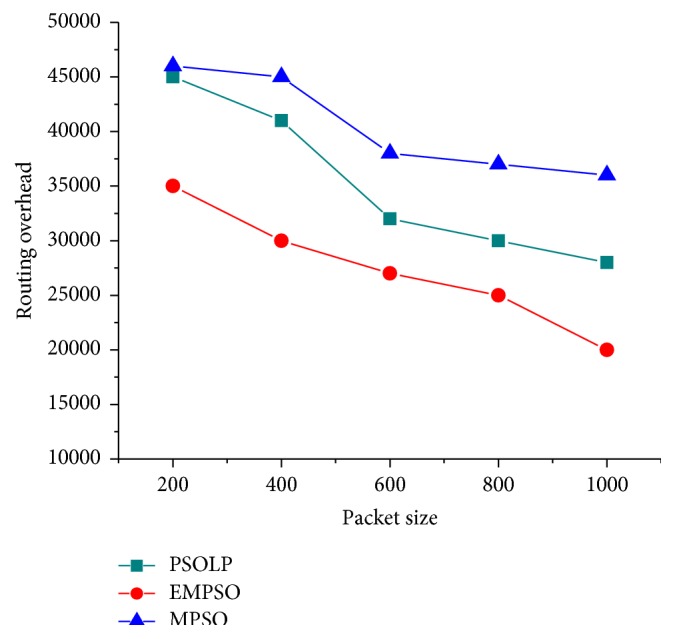
Routing overhead versus packet size.

In this simulation, the routing overhead was evaluated for EMPSO, PSOLP, and MPSO while varying the packet size with different stages.  Figure 7 depicted the routing overhead that occurred during the routing process. EMPSO has 2.13% lower overhead when compared with PSOLP and MPSO; it reveals in reduced to generate control routing packets and hence alternate routing will be triggered. Therefore, the routing overhead is decreasing. In PSOLP and MPSO, the control packet flooded throughout the network until the destination was reached to find the loop-free routing. It caused the routing overhead. The routing overhead caused by EMPSO performs less than the other two protocols because of its PSO mechanism, a limited exchange of routing control messages among the nodes for the route discovery phase. EMPSO had a minimum routing overhead of approximately 43% when an optimal path of the nodes was selected.

### 4.5. Energy Consumption

**Figure 8:**
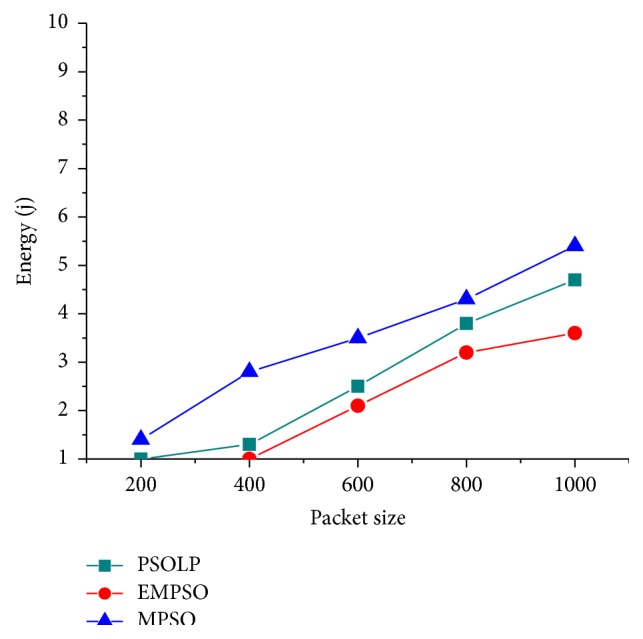
Energy consumption versus packet size.

Figure 8 shows the energy consumption for three techniques when the packet size is increased from 250 to 1000 bytes. It is clearly seen that EMPSO has 0.28% less energy consumption when compared to related schemes since it considers reliability measures by CRNN mechanism for finding multipath. Figure 8 shows the total energy consumption of a network while varying packet size. It is noticed that the EMPSO consumes minimum energy about 3.22 j while varying the packet size. PSOLP and MPSO have larger energy consumption because they tend to establish longer routes and to find as many nodes-disjoint routes as possible in a route discovery attempt. The other two related schemes such as PSOLP and MPSO do not consider the energy factor, and also they required more control packets to establish routing that causes more energy consumption. The proposed EMPSO selects the optimal path that reveals efficient solution with less energy consumption. In the proposed EMPSO, the energy consumption of a node increases slightly as the number of nodes also increases.

### 4.6. Path Optimality

**Figure 9:**
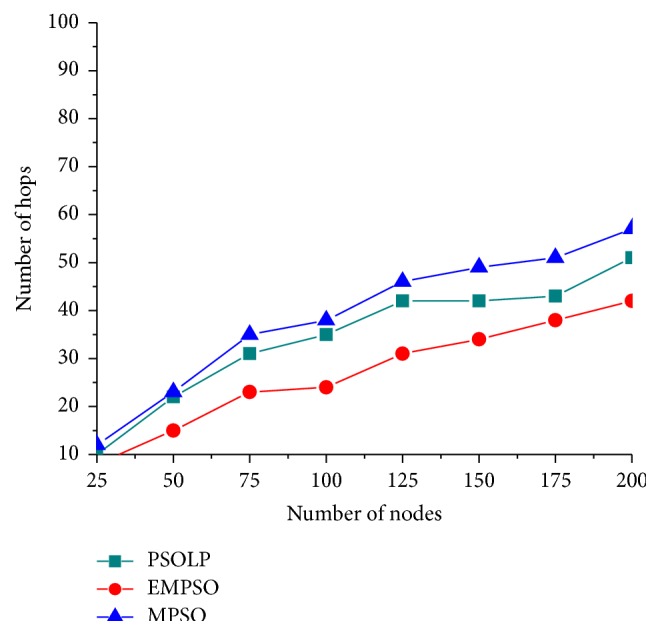
Number of hops versus number of nodes.

Figure 9 shows the path optimality of the different routing protocols. It is noticed that the EMPSO used minimum hops to forward the packets from source to destination because the EMPSO protocol uses the PSO algorithm for constructing the loop-free routing between source and destination. This algorithm achieves the minimum number of hops due to PSO mechanism. This leads to elimination of faraway nodes. So the amount of hops is minimized. As a consequence of this, the average hop count is reduced in the proposed EMPSO scheme. Thus the proposed EMPSO scheme achieves path optimality compared with related scheme.

## 5. Conclusion

The prime objective of the proposed EMPSO scheme is to develop energy-aware multipath routing based on particle swarm optimization in mobile ad hoc networks. Development of reliable routing with the use of PSO is conversed. The proposed EMPSO scheme has used a PSO mechanism to reveal the optimal route to minimize the routing overhead and ensure the reliability in MANET. A novel scheme of energy-aware multipath routing based on particle swarm optimization is developed for ensuring reliable routing in MANET. CRNN is used to find multipath among nodes using reliability measures such as transmission cost, energy factor, and optimal traffic ratio. A packet can be forwarded through a selected optimal path. This is a novel way to enhance reliability in routing among nodes. It emphasizes ensuring reliable routing during data transmission when it follows the PSO mechanism. Simulation results show that the proposed EMPSO scheme reveals good results compared with related schemes. It is concluded that the proposed EMPSO based multipath routing can be a potential solution for real time multimedia applications. Future research direction is to use simulated annealing technique to enhance energy efficiency.

## Figures and Tables

**Table 1:** Parameter settings for simulation.

Parameter	Value
Simulation area	1000 × 1000 m
Simulation time	1000 sec
Number of nodes	50, 100, 150, and 200
Transmission range	200 m
Speed	0–50 m/sec
Movement model	Random waypoint model
Traffic type	CBR/UDP
Packet size	1000 bytes
Rate	250 kb/s
Pause time	500 sec
